# Monoamine oxidases: A missing link between mitochondria and inflammation in chronic diseases ?

**DOI:** 10.1016/j.redox.2024.103393

**Published:** 2024-10-11

**Authors:** Lise Beucher, Claudie Gabillard-Lefort, Olivier R. Baris, Jeanne Mialet-Perez

**Affiliations:** Univ Angers, Inserm, CNRS, MITOVASC, Equipe MitoLab, SFR ICAT, Angers, F-49000, France

**Keywords:** Inflammation, Chronic diseases, DAMPs, Mitochondria, Oxidative stress, Monoamine oxidases

## Abstract

The role of mitochondria spans from the regulation of the oxidative phosphorylation, cell metabolism and survival/death pathways to a more recently identified function in chronic inflammation. In stress situations, mitochondria release some pro-inflammatory mediators such as ATP, cardiolipin, reactive oxygen species (ROS) or mitochondrial DNA, that are believed to participate in chronic diseases and aging. These mitochondrial Damage-Associated Molecular Patterns (mito-DAMPs) can modulate specific receptors among which TLR9, NLRP3 and cGAS-STING, triggering immune cells activation and sterile inflammation. In order to counter the development of chronic diseases, a better understanding of the underlying mechanisms of low grade inflammation induced by mito-DAMPs is needed. In this context, monoamine oxidases (MAO), the mitochondrial enzymes that degrade catecholamines and serotonin, have recently emerged as potent regulators of chronic inflammation in obesity-related disorders, cardiac diseases, cancer, rheumatoid arthritis and pulmonary diseases. The role of these enzymes in inflammation embraces their action in both immune and non-immune cells, where they regulate monoamines levels and generate toxic ROS and aldehydes, as by-products of enzymatic reaction. Here, we discuss the more recent advances on the role and mechanisms of action of MAOs in chronic inflammatory diseases.

## Highlights

• Sterile inflammation underlies the development of age-associated chronic diseases.
• Mitochondria participate in the activation of inflammation pathways and immune processes through the release of mito-DAMPs.
• Monoamine oxidase (MAOs) are activated in several chronic diseases, leading to altered catecholamines and serotonin levels and/or accumulation of H_2_O_2_ and aldehydes.
• Recent evidences link MAOs activation to the regulation of immune cells and inflammatory processes.
• The selective MAO inhibitors currently used for depression could relieve inflammatory processes in chronic diseases.

## Graphical abstract

Mitochondria has emerged as an important regulator of sterile inflammation in chronic diseases, through the release of pro-inflammatory mediators called mito-DAMPs (Damage-Associated Molecular Patterns). Recently, monoamine oxidases (MAO), the mitochondrial enzymes that degrade catecholamines and serotonin, were described as potent regulators of chronic inflammation in obesity-related disorders, cardiac diseases, cancer, rheumatoid arthritis and pulmonary diseases. The role of these enzymes in inflammation embraces their action in both immune and non-immune cells, where they regulate monoamines levels and generate toxic ROS and aldehydes, as by-products of enzymatic reaction. This review discuss the more recent advances on the role and mechanisms of action of MAOs in chronic inflammatory diseases.

**Figure:**
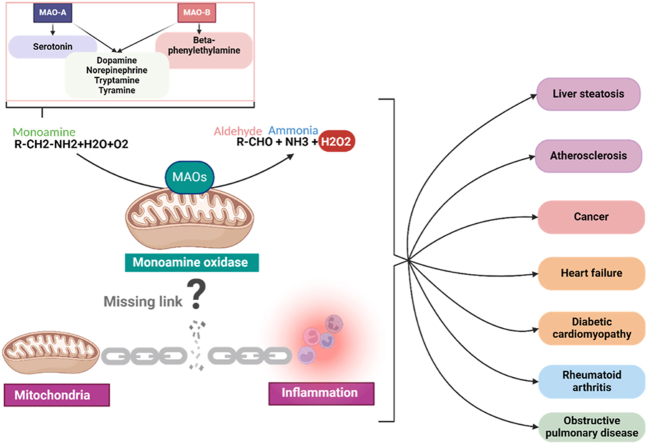

## 1 Introduction: Sterile inflammation and chronic diseases

Inflammation is a reaction of the body to pathogen infection or tissue injury through the recognition of PAMPs (pathogen-associated molecular patterns) or DAMPs (cell damage-associated molecular patterns), respectively [1]. DAMPs are endogenous molecules released upon cell stress or death that initiate a reaction called “sterile inflammation” in order to drive tissue repair and regeneration. This complex defence strategy is orchestrated by the immune system and involves sequential and coordinated response of different immune cells such as macrophages, leucocytes, dendritic cells (DCs), mast cells or natural killer (NK) cells. If unresolved, the inflammatory response becomes chronically activated and can lead to further tissue damage. Sterile inflammation participates in the development and progression of many chronic diseases such as post-myocardial infarction remodelling, atherosclerosis, diabetes, rheumatoid arthritis, liver steatosis and cancer [2].

The major mediators of chronic inflammation are cytokines and interferons (IFN), which are produced in response to DAMPs released by injured cells. The cell receptors that sense and recognize DAMPs belong to the family of Pattern Recognition Receptors (PRRs) present on both immune and non-immune cells [3]. Among the different classes of PRRs are Toll-like receptors (TLRs), retinoic acid-inducible gene I (RIG-I)-like receptor (RLR), nucleotide-binding oligomerization domain (NOD)-like receptor (NLR), C-type lectin receptors (CLRs) and multiple intracellular DNA sensors [4]. Once activated, PRRs initiate downstream signalling pathways of transcription factors such as nuclear factor-κB (NF-κB), mitogen-activated protein kinase (MAPK) and type I-interferon regulatory factor (IRF), resulting in upregulation of pro-inflammatory cytokines and IFN, that turn on and boost inflammatory processes [5]. This priming step is followed by the amplification of the inflammatory process through the binding of cytokines, chemokines and IFN on their cognate receptors, leading to the recruitment of neutrophils and macrophages and the production of other inflammatory mediators and the induction of T cell-mediated adaptive immune response [1].

Inhibition of chronic inflammation has shown beneficial effects in a broad spectrum of diseases, ranging from rare autoinflammatory diseases to common conditions such as rheumatoid arthritis, type II diabetes, atherosclerosis, and myocardial infarction [6]. For example, blockade of IL-1R with anakinra can relieve post-myocardial inflammation and remodelling or reduce the severity of type II diabetes [7,8]. In addition, inhibition of IL-1R signaling pathway can decrease the pro-inflammatory environment around the tumor, relieving the risk of tumorigenesis and metastasis in cancer [9]. Thus, targeting the immune system holds promise to counter the development and progression of chronic diseases. This calls for a better understanding of important signaling pathways regulating inflammatory response. In this review, we will focus on mitochondria-driven inflammatory pathways with a particular emphasis on monoamine oxidases (MAOs) as new regulators of inflammation. We will provide an overlook on the role of MAOs, either as a catecholamine/serotonin consumers or ROS producers in different pathophysiological contexts.

## 2 The mitochondria/ROS axis in inflammatory signaling pathways

### 2.1 Mitochondrial DAMPs

DAMPs released during cell damage can be nucleic acids, proteins, ions, glycans or metabolites. They originate from different cell compartments such as extracellular matrix (Tenascin C, Proteoglycan), nucleus (histone, HMGB1), cytoplasm (S100 proteins) and mitochondria (ATP, cytochrome C, mitochondrial DNA, N-formyl peptides, cardiolipin) [10]. Mitochondria are the main organelles to release various types of DAMPs in the cytosol, cell surface, or extracellular space. Due to their endosymbiotic origin, some of their molecules and structures share many features with bacteria, suggesting that mitochondrial DNA (mtDNA) and proteins act in a similar way as bacterial PAMPs. Indeed, it is believed that mtDNA, mtRNA, cardiolipin, N-formyl peptides, succinate and ATP are very potent stimulators of inflammation [11]. In addition, mitochondria are the main site of reactive oxygen species (ROS) production, which is central to the progression of pro-inflammatory chronic diseases [12,13]. Accumulation of cellular ROS, termed oxidative stress, favours the release of DAMPs through cell injury [14]. ROS can also be directly released by stressed or dead cells in case of tissue injury, acting themselves as DAMPs, to promote secretion of inflammatory mediators [15,16]. Finally, ROS can also be produced at high levels by immune cells such as monocytes/macrophages, which sustains tissue injury in a vicious circle termed “ROS-induced ROS release” [17]. Antioxidants such as ROS scavengers or antioxidative enzymes can inhibit inflammation induced by several DAMPs [18,19]. A master regulator of the antioxidant response is the Kelch-like ECH-associated protein 1 (Keap1) - nuclear factor erythroid 2-related factor 2 (Nrf2) pathway, which regulates the transcription of anti-oxidant genes. Keap1-NRF2 system plays a pivotal role in attenuating inflammation [17]. Therefore, mitochondrial ROS act alone or in synergy with other DAMPs to regulate the pro-inflammatory response. Mito-DAMPs-driven inflammation is highly specific to cellular stress responses and may contribute to tissue-specific pathologies.

### 2.2 Signaling pathways activated by mito-DAMPs and ROS

**Fig. 1:**
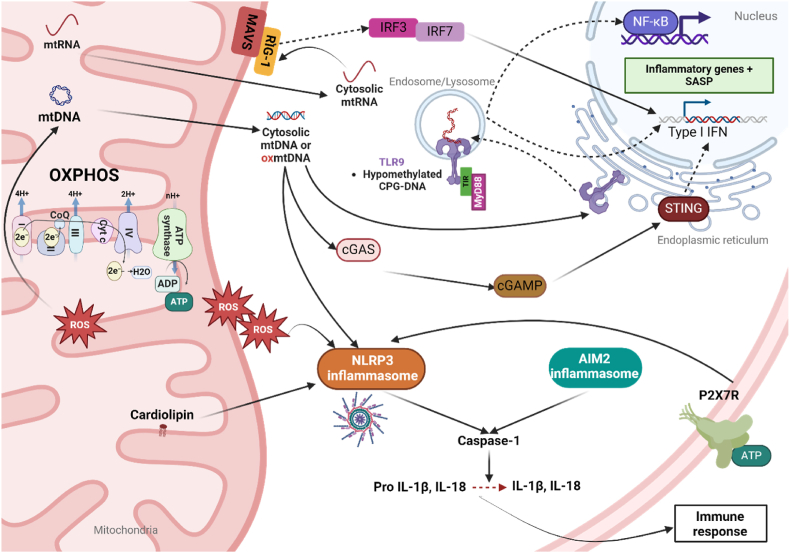
Signaling pathways activated by mito-DAMPs. Upon mitochondrial damage, mito-DAMPs such as mtDNA, mtRNA, cardiolipin or ATP are able to stimulate cellular inflammatory pathways. Mitochondrial nucleic acids (mtDNA and mtRNA) are released into the cytoplasm, where they activate several cytosolic immune sensors. Cytosolic mtRNA is detected by Retinoic acid-Inducible Gene I (RIG-I), which activates Mitochondrial Antiviral-Signaling protein (MAVS), leading to the activation of Interferon Regulatory Factors 3 and 7 (IRF3 and IRF7) and the subsequent induction of a type I interferon (IFN) response. Cytosolic mtDNA activates cyclic GMP-AMP synthase (cGAS), which catalyzes the production of cyclic GMP-AMP (cGAMP). cGAMP binds to and activates the STING pathway, leading to the transcription of IFN and inflammatory genes via NF-κB. mtDNA can also be recognized by Toll-like receptor 9 (TLR9) within the endosomal/lysosomal compartment. Upon binding to hypomethylated CpG-DNA, TLR9 triggers downstream signaling cascades involving MyD88, ultimately leading to the expression of inflammatory genes. Oxidized mtDNA or cardiolipin can also activate NLRP3 and AIM2 inflammasomes, leading to the recruitment and activation of caspase-1. Activated caspase-1 cleaves pro-IL-1β and pro-IL-18 into their mature forms, which are then secreted, promoting an immune response. ATP through P2X7R is also a potent activator of NLRP3 inflammasome.

Several classes of PRRs can be activated in response to mito-DAMPs, initiating the immune response in both immune and non-immune cells. The downstream effectors and signaling pathways activated by mito-DAMPS are illustrated in Fig. 1.

TLR9 was the first identified nucleic acid sensing TLR that recognizes hypo-methylated cytosine-guanosine (CpG) motifs present on bacterial or viral DNA, but also on mtDNA [20]. Under basal conditions, TLR9 is located in the endoplasmic reticulum (ER), and upon stimulation by inducers, it translocates to the membrane of endosomes or lysosomes to initiate inflammation through myeloid differentiation primary response 88 (MyD88)-mediated IFN and cytokine expression [11]. In the lung, mtDNA can amplify airway inflammation in allergic mice through TLR9 [21]. In the heart, TLR9 mediates inflammation and heart failure due to insufficient degradation of mtDNA by autophagy during pressure overload [22,23]. As recently demonstrated, another sensor RIG-I can respond to mitochondrial RNA (mtRNA) to activate IFN and pro-inflammatory cytokines through interaction with the mitochondrial antiviral signaling protein (MAVS) [24] (Fig. 1).

The cGAS-STING signaling axis is also a critical regulator of DNA-dependent IFN response that was recently identified [[25], [26], [27]]. The enzyme cyclic guanosine monophosphate–adenosine monophosphate (cGAMP) synthase (cGAS), detects intracellular DNA and generates the second messenger cGAMP, which activates the ER-resident adaptor Stimulator of Interferon Genes (STING) [28]. Activated STING engages TANK-binding kinase 1 (TBK1), which phosphorylates IRF3 and promotes the expression of IFNβ and interferon-stimulated genes (Fig. 1). Release of mtDNA into the cytosol is a prominent trigger of cGAS-STING, which might be further enhanced by oxidative stress and oxidative damage to mtDNA [29]. The activation of cGAS-STING by mtDNA is also an important driver of senescence-associated secretory phenotype and inflammation during aging [30]. It also participates in many inflammatory diseases [31]. Interestingly, a recent screening performed by He et al. identified some key regulators of mtDNA release and cGAS-STING-mediated inflammation as components of mitochondria cristae architecture, including cardiolipin, OPA1, MICOS, prohibitin [32].

Among the family of intracellular PRRs, NLRP3 or AIM2 form multiprotein complexes called inflammasomes, with the adaptor apoptosis-associated speck-like (ASC) protein and procaspase-1. Inflammasome assembly prompts caspase-1 expression and upholds discharge of proinflammatory cytokines IL-1β and IL-18, through cleavage of pro-IL-1β and pro-IL-18 [18] (Fig. 1). Mounting evidence implicates mitochondria in NLRP3 activation. Cardiolipin, a phospholipid found in inner mitochondrial membranes, has been demonstrated to bind to NLRP3 *in vitro* after translocation to the outer membrane [33], and to promote NLRP3 oligomerization [34]. ATP was one of the first described NLRP3 inflammasome activators. It can be released at high concentrations in the extracellular space during tissue damage such as ischemia-reperfusion by many different cell types, including cardiomyocytes, cardiac fibroblasts, endothelial cells, macrophages and erythrocytes. ATP acts as a DAMP by activating NLRP3 inflammasome upon P2X7 membrane receptor binding and K^+^ efflux [35]. Resulting cleavage of Caspase-1 induces IL-1β secretion and subsequent immune response [36]. In respiratory diseases such as chronic obstructive pulmonary disease (COPD), smokers had elevated ATP concentrations in bronchoalveolar lavage fluid (BALF), which potentiated the response of airway macrophages to promote secretion of pro-inflammatory and tissue-degrading mediators such as MMP9 Mitochondrial metabolites have also been described as important modulators of inflammatory pathways in innate immune cells (i.e. dendritic cells and macrophages)[37]. Key tricarboxylic acid intermediates such as succinate can modulate the production of IL-1β via the NLRP3 inflammasome [38]. Most importantly, among DAMPs, mtDNA has emerged as a major trigger of the inflammasome. Nakahira et al. first linked mtDNA to NLRP3 inflammasome activation, showing that LPS promoted cytosolic mtDNA accumulation in macrophages to augment caspase-1 activation and IL-1β/IL-18 secretion [39]. Interestingly, this response required ROS as an upstream activator of NLRP3, which was suggested to enhance mtDNA release into the cytoplasm. NLRP3 also appears to bind oxidized mtDNA species with higher affinity [40] and additional reports have implicated oxidized mtDNA in NLRP3 inflammasome activation [41,42]. The crystal structure of NLRP3 contains a disulfide bond between Cys-8 and Cys-108, which is highly sensitive to oxidative stress [43]. In addition, it has been shown that mitochondrial complex I inhibitor rotenone or complex III inhibitor antimycin increased mtROS production and, as a result, increased NLRP3 activation [42]. Altogether, this is indicative of a crucial redox regulation of NLRP3 (Table 1). Along NLRP3, caspase 1 can also be regulated by Cys oxidation and gluthationylation [44]. The inflammasome is not the only effector of mito-DAMPs that is sensitive to oxidation, as referenced in Table 1. Some DAMPs are also known to be regulated by oxidation such as Cyt *c*, the proteins S100, HMGB1 [45,46], along with other major regulators of inflammation, such as Keap1/NRF2, NFκB and its inhibitor IκB, p38 MAPK [45], IRF3 [47], Myd88 [48], STING [49] and the RNA sensor RIG1 and MAVS [15,50].

**Table 1:** Redox modifications of inflammation factors.

Redox Modif.	Tyr nitration	S- Nitrosylation	Met Sulfoxidation	S-glutathionylation	Cys-oxidation
**Modified Proteins**	Cyt *c*	Cyt *c*	Cyt *c*	S100A9	S100A9
	NF-κB p65	S100A8	RIG-1	IRF3	Nrf2
	p38 MAPK	NF-κB p50	S100A9	NF-κB p50	HMGB1
		Keap1		IκBα, IκBβ	Keap1
		NLRP3		Casp1	Casp1
					STING
					MyD88
					MAVs
					NF-κB p50

At present, although ROS are important mediator of immune signaling, the main source of mitochondrial ROS is still a matter of debate. Several enzymatic systems have been identified such as electron transport chain, NADPH oxidases, p66shc or monoamine oxidases (MAOs), that we will review in detail.

## 3 Monoamine oxidases as putative mediators of inflammatory pathways through biogenic amines degradation and/or ROS production

### 3.1 Monoamine oxidases: genes, isoforms and biochemistry

MAOs are flavin oxidases located at the outer membrane of mitochondria and anchored through their Cterminal domain [51]. They are ubiquitous enzymes encoded by two distinct genes, MAO-A and MAO-B, located on the X chromosome [52]. Their main function consists in the regulation of biogenic amines levels such as norepinephrine (NE), dopamine (DA), serotonin (5-HT) and tyramine in the body [51]. In order to be degraded inside the cells, neurohormones are internalized by membrane transporters of two different types: non-selective transporters such as OCTs or selective transporters such as norepinephrine transporter (NET), dopamine transporter (DAT) or serotonin transporter (SERT) [53,54].

MAO-A and MAO-B share ∼70 % amino-acid identity but have distinct selectivity for substrates and inhibitors [51,53]. MAO-A preferentially catalyzes the oxidative deamination of 5-HT while MAO-B is more specific for phenylethylamine and benzylamine. Both isoforms can degrade NE, DA and tyramine. The first generation of MAO inhibitors was irreversible and non-selective, such as pargyline, iproniazid, and phenelzine [55]. Later on, the introduction of selective inhibitors for MAO-A (clorgyline) or MAO-B (selegiline or deprenyl, rasagiline, safinamide) has allowed a better understanding of the roles of MAO-A versus MAO-B in the different brain areas and in peripheral tissues [51]. Some selective and reversible inhibitors such as moclobemide for MAO-A were further introduced to avoid the side effects observed with some irreversible inhibitors that were associated with hypertensive crisis due to tyramine overload [56]. Owing to the crucial role played by MAOs in the deactivation of neurohormones, MAO-related disorders are frequently associated with psychiatric and neurological issues. Some MAO inhibitors are currently used for the treatment of atypical depression (moclobemide) or reduction of motor symptoms in Parkinson's disease (rasagiline, safinamide, selegiline) [57]. Apart from neurological disorders, MAO enzymes are also significantly involved in cardiac and vascular diseases, metabolic and renal diseases and cancer [58].

### 3.2 Role of MAOs in the regulation of 5-HT and catecholamines turn-over: consequences on inflammation

Most interestingly, some recent publications now put forward a role for MAOs in the regulation of inflammatory process through the turnover of NE, DA and 5-HT. During inflammation, some cross-talks between the autonomic nervous system and the immune system have been established, along with the presence of biogenic amines in different types of immune cells [[59], [60], [61]]. A complete pathway of catecholamine biosynthesis and degradation by MAOs was observed in T cells [62,63] as well as macrophages and neutrophils [60,64]. MAOs regulate T cells function through catecholamines degradation [63]. Additionally, serotonin has been known for a long time to act as a regulator of inflammatory processes, and macrophages and lymphocytes also possess a full 5-HT system with 5-HT synthases and MAO-A [65,66].

### 3.3 MAOs as sources of DAMPs through the generation of toxic by-products?

**Fig. 2:**
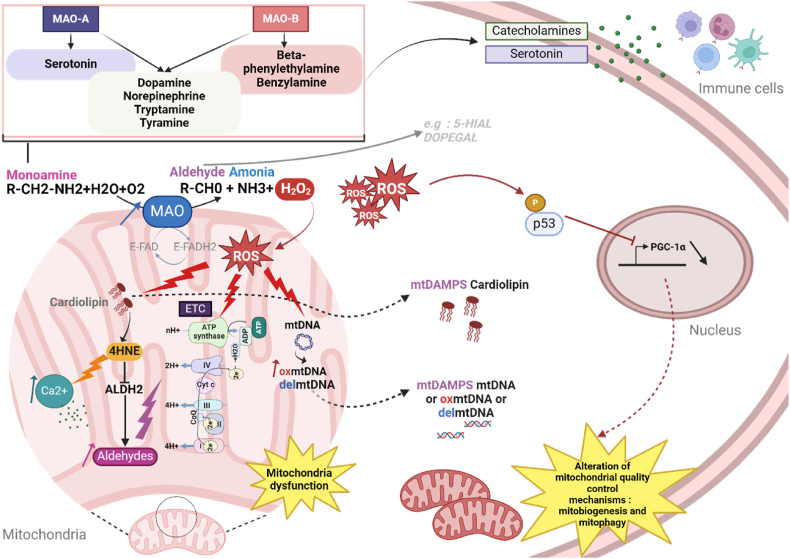
MAOs as sources of DAMPs through the generation of toxic byproducts ? Monoamine oxidases (MAOs) A and B are located in the mitochondrial outer membrane. MAOs metabolize monoamines in an oxidative deamination reaction, resulting in the generation of aldehydes, ammonia and H_2_O_2_ as degradation by-products. H_2_O_2_ a reactive oxygen species (ROS), can enter the mitochondria and cause cardiolipin peroxidation, which is a putative DAMP. Cardiolipin peroxidation can generate 4-hydroxynonenal (4-HNE), which in turn inhibits the activity of aldehyde dehydrogenase 2 (ALDH2), allowing further accumulation of aldehydes in the mitochondria and alteration of the electron transport chain. Inhibition of ALDH2 also results in mitochondrial calcium (Ca^2+^) overload. ROS generated by MAOs can also lead to the oxidation/deletion of mitochondrial DNA (mtDNA). MtDNA can escape the mitochondria and activate cellular inflammation signalling pathways. MAO-dependent ROS can also cause downregulation of nuclear peroxisome proliferator-activated receptor gamma coactivator 1 alpha (PGC-1α). This will ultimately lead to impaired mitochondrial quality control, mitochondrial biogenesis and mitophagy. Catecholamines and serotonin can also be degraded and regulate the activity of immune cells.

In addition to regulating neurohormone concentrations, MAOs generate toxic byproducts that contribute to a number of deleterious effects related to tissue injury and chronic inflammation [58]. MAOs convert biogenic amines into their corresponding aldehydes and generate stoichiometric amounts of H_2_O_2_ and ammonia (Fig. 2). An overactivation of MAOs, due to substrate overload or protein upregulation, favours the accumulation of H_2_O_2,_ leading to oxidative stress in different pathophysiological situations [67]. Any condition leading to the activation of the sympathetic nervous system (SNS) will increase the release of catecholamines that serve as substrates for the different MAO isoforms. Such chronic activation of the SNS is observed in hypertension, cardiovascular diseases, obesity and aging [68]. Some mental stress conditions are also associated with chronic SNS activation, leading to oxidative stress and inflammation, as exemplified with the traffic noise exposure [69]. 5-HT is released by activated platelets in the periphery and will be released in conditions leading to platelet activation such as ischemia [70]. On the other hand, aldehydes are highly reactive and toxic compounds that contain a terminal carbonyl group. They are detoxified by conjugation to glutathione (GSH) or by catabolism through aldehyde dehydrogenases (Aldh) [71]. MAOs can generate aldehydes directly through 5-HT or catecholamine degradation, such as 5-hydroxyindoleacetaldehyde (5-HIAL) or catecholaldehyde 3,4-dihydroxyphenylglycolaldehyde (DOPEGAL), respectively, that are involved in neurotoxicity [72] or diabetic cardiomyopathy [73]. Aldehydes such as 4-HNE or MDA can also be formed indirectly through ROS-mediated peroxidation of polyunsaturated fatty acids in response to MAOs activation [74].

Interestingly, mounting evidences now show that MAO overactivation affects primarily the mitochondria. MAO activation leads to H_2_O_2_ build-up inside the organelle. The time-course of redox changes in cardiac myocytes expressing a mitochondria-targeted H_2_O_2_ fluorescent probe (HyPer-mito) showed an increase after only 10 min of dopamine addition, whereas the cytosolic probe (HyPer-cyto) increased after 30 min [75]. Furthermore, Santin et al. confirmed the accumulation of H_2_O_2_ in the mitochondria with a MitopY1 probe, following application of MAO substrate on adult cardiomyocytes. Interestingly, inhibition of MAO-A or MAO-B in cardiomyocytes has a strong effect on global ROS inhibition, suggesting that some redox crosstalk exists between different ROS sources, leading to an amplification mechanism [53]. Initial production of ROS could trigger the activation of secondary ROS sources. Such mechanism has already been described between NADPH oxidases and mitochondrial ROS production, through the depolarization of mitochondrial membrane potential [76,77]. ROS-induced lipid peroxidation and aldehydes generation also constitute a deleterious amplification mechanism for the mitochondria. Interestingly, mouse adult cardiomyocytes treated with the MAO substrate tyramine displayed mitochondrial 4-HNE accumulation resulting from H_2_O_2_-mediated cardiolipin peroxidation [74]. This mitochondrial accumulation of 4-HNE was particularly deleterious to the heart as stimulation or overexpression of mitochondrial Aldh2 prevented mitoCa^2+^ overload, mitochondrial damage and cardiac dysfunction due to MAO-A activation [74]. Thus, the generation of toxic byproducts by MAO is principally damaging for mitochondrial components, impairing oxidative phosphorylation, mitochondrial membrane potential and mitoCa^2+^ homeostasis [74,75,[78], [79], [80], [81]]. In the long term, mitochondrial quality control mechanisms can also be impaired such as mitochondrial biogenesis with PGC-1α downregulation and mitophagy [82,83]. Altogether, these data suggest that MAOs could represent a driving force in cardiolipin or mtDNA oxidation in stress conditions. For instance, some studies showed that MAO-A promoted the accumulation of the DNA oxidation marker 8-OH-dG outside of the nucleus, suggesting preferential oxidation of mtDNA [83,84]. In addition, it has been shown that catecholamine metabolism, which involves both MAOs, is detrimental for mtDNA integrity in dopaminergic neurons and adrenal gland [85,86]. Overexpression of MAO-A or MAO-B in differentiated dopaminergic-like SH-SY5Y neuroblastoma cells leads to enhanced generation of mtDNA deletions, which is further exacerbated in the presence of dopamine. Moreover, overexpression of MAO-B alone specifically in astrocytes in mouse leads to dramatically increased mtDNA deletion loads in the susbtantia nigra [85].

While these data establish a link between MAO activation and the oxidation of mitochondrial components, the role of MAOs in inflammatory diseases is just beginning to be unraveled. MAO activation could regulate inflammatory pathways through the regulation of catecholamine/5-HT-mediated activation of immune cells or through the release of mito-DAMPs.

## 4 Regulation and function of MAOs in immune cells

Some interesting findings have been described on the regulation of MAO expression and function in the context of chronic inflammation and in different immune cells subsets.

### 4.1 Upregulation of MAOs during chronic inflammation

The activity and expression of MAOs have been shown to be regulated in pro-inflammatory situations. In the model of inflammation induced by LPS injection in rats, an upregulation of MAO expression was observed in the vasculature and MAO inhibitors treatment decreased oxidative stress and vascular dysfunction in this context [87]. In another rat model of periodontal disease induced by LPS, MAO-B was one of the most upregulated genes and MAO inhibitors significantly counteracted LPS-associated elevation of H_2_O_2_ and TNF-α [88]. Moreover, in the model of LPS-induced endotoxemia, genetic deletion of MAO-B or pharmacological inhibition of MAO-B with rasagiline decreased the number of inflammatory cells into the peritoneal cavity and reduced IL-1β plasma levels [89]. Depression can also be induced by LPS in rats and inhibition of MAO-A/MAO-B with tranylcypromine decreased LPS-induced expressions of IL-1β, IL-6, TNF-α and IFN-γ in the brain [90]. In renal ischemia/reperfusion, an increase in MAO activity occurs in the first minutes of reperfusion, due to the massive release of 5-HT and catecholamines, leading to ROS production and release of inflammatory cytokines [91,92]. In those different contexts of chronic inflammation, MAOs are overexpressed both in non-immune and immune cells where they can act simultaneously. They can also act in a paracrine manner, as demonstrated in the heart with a cross-talk between cardiomyocytes, mesenchymal stem cells and monocytes [93]. Although it is difficult to decipher the respective roles of MAOs in different cell types, some recent studies have provided convincing evidences that MAOs are indeed key regulators of immune cells function.

### 4.2 Regulation and function of MAOs in macrophages

Macrophages are large, specialized cells that rapidly recognize, engulf and destroy pathogens or apoptotic cells. In response to tissue injury, they change their profile and adopt a pro-inflammatory phenotype finely tuned by specific cytokines and growth factors. Early studies demonstrated for the first time a complete pathway of catecholamine biosynthesis and degradation by MAOs in macrophages, activated during inflammation to act in a paracrine/autocrine manner [60]. Stimulation of macrophages with LPS led to upregulation of MAO-A enzyme expression [60].

The functional importance and the role of MAOs in macrophages has been established more recently. In U937 macrophages, the application of safinamide, a selective MAO-B inhibitor, reduced the secretion of pro-inflammatory cytokines, chemokines and matrix-metalloproteases in response to LPS through inhibition of NF-κB/TLR4 signaling pathway [94]. In another study, LPS/ATP stimulation of mouse bone-marrow-derived macrophages (BMDMs) upregulated MAO-B protein expression and activity and inhibition of MAO-B with rasagiline decreased ROS production, NF-κB activation, NLRP3 expression and IL-1β secretion [89]. Additionally, macrophages can upregulate a full 5-HT system with 5-HT synthases and MAO-A when exposed to pro-inflammatory signaling, leading to NF-κB activation and the release of inflammatory cytokines [66]. On the other hand, a study evaluated the response of monocyte/macrophages to anti-inflammatory Th2 cytokines IL-4 and IL-13 and found that MAO-A was one of the most upregulated genes in a panel of genes, while MAO-B was unchanged [95,96]. The functional role of MAO-A upregulation in this context was not established, but the authors suggested a novel role of the enzyme in the resolution of inflammation in M2 macrophages [97,98]. Overall, it is possible that MAOs could have different effects on macrophages, depending on the tissue or the pathological context. In adipose tissue, it was recently shown that adipose tissue macrophages (ATMs) could upregulate the expression of MAO-A during aging, with potential implications in catecholamine regulation and adipocytes function [99]. In addition, upregulation of MAO-A expression in tumor-associated macrophages is observed in mice inoculated with B16-OVA melanoma tumors which leads to the inhibition of antitumor immunity [100].

### 4.3 Regulation and function of MAOs in lymphocytes, neutrophils and other immune cells

The presence of MAOs in circulating lymphocytes and neutrophils has been described for a long time, with a preferential expression of MAO-A compared to MAO-B [65,101]. Following T cell activation with concanavalin-A, MAO-A expression increased robustly and its inhibition reduced proliferation [63]. Mast cells are a cell lineage of hematopoietic stem cells responsible for allergy and inflammation that express MAO-B [102]. MAO-A expression in dendritic cells is upregulated upon alcohol treatment compared to untreated DCs by elevation of cAMP levels, which decrease concentration of 5-HT in the extracellular medium and cause neurological and immunological deregulation [103].

## 5 Pathophysiological evidences on the role of MAO in chronic inflammation

Monoamine oxidases have recently emerged as important mediator of inflammation and immunity through the regulation of serotonin/catecholamine levels or through oxidative stress mechanisms. Here, we will provide an overview on the most recent findings highlighting the mechanisms of action of MAOs in inflammation associated with obesity-related disorders, cancer, cardiac diseases, rheumatoid arthritis and obstructive pulmonary disease.

### 5.1 Pro-inflammatory role of monoamine oxidases in obesity-related disorders

• **Role of macrophage MAO-A in adipose tissue homeostasis**

In adipose tissue, sympathetic nerves release catecholamines and stimulate lipolysis through the activation of β-adrenergic receptors in adipocytes [104]. This pronounced effect of NE generates energy substrate through the hydrolysis of triglycerides, particularly in fasting periods. However, this effect declines with age and in obese subjects, leading to excessive accumulation of fat and chronic inflammation. In this context, an unexpected function of MAO-A in adipose tissue homeostasis has recently been uncovered [99]. MAO-A represents about 70–80 % of the total MAO activity in human adipose tissue [105]. In their study, Camell et al. demonstrated that in aged mice, a strong upregulation of MAO-A expression in ATMs was responsible for the excessive degradation of NE, thus preventing NE-activation of lipolytic signaling [99]. Interestingly, MAO-A upregulation in aged ATMs was linked to the activation of NLRP3 by DAMPs and the subsequent upregulation of Growth Differentiation factor 3 (GDF3) [99]. This study highlighted the importance of a close proximity of sympathetic nerves to ATMs in the regulation of lipolysis by catecholamines/MAO-A. However, the putative consequences of the enhanced degradation of NE by MAO-A on the phenotype and function of ATMs were not studied in the context of aging. On the other hand, in obesity, Pirzgalska et al. recently explored the specific role of NE in ATMs regulation [106]. ATMs are among the predominant immune subtypes that accumulate in obese mice and humans [107]. They display a pro-inflammatory phenotype and are believed to play a major role in adipose tissue dysfunction through chronic inflammation [108]. Surprisingly, Pirzgalska et al. identified a new subpopulation of ATMs in close contact with sympathetic neurons that they called SAMs for sympathetic neuron-associated macrophages. Transcriptional profiling indicated that SAMs possessed the full machinery to import and degrade NE through the NET and MAO-A, contrary to other macrophages [106]. They further showed that stimulation of sympathetic nerve activity led to the release of NE and its import into SAMs, which increased their pro-inflammatory profile. Finally, in two different models of obesity in mice, SAMs accumulated and displayed pro-inflammatory profiles while inhibition of NE import and degradation in SAMs ameliorated obesity, thermogenesis and lipolysis [106]. Very recently, some further mechanistic insights linking NE/MAO-A signaling to macrophages function in the context of obesity have been uncovered [109]. The authors observed that Allograft inflammatory factor-1 (AIF-1) deficiency, a protein preferentially expressed in myeloid cells, conferred strong protection against obesity induced by HFD, insulin resistance and increased energy expenditure in mice. Aif1^−/−^ mice had enhanced thermogenic gene expression, increased β−AR signaling and higher levels of NE in adipose tissue, with a nearly complete extinction of MAO-A protein and activity in ATMs. Additionally, AIf1^−/−^ macrophages of epididymal adipose tissue displayed M2-like phenotype accompanied by lower levels of IL-6 and IL-12p70 [109]. Finally, in adipose tissue from obese patients, AIF1 expression correlated with MAO-A expression, further supporting the link between AIF1 and MAO-A in NE catabolism in macrophages, obesity, insulin resistance and glucose intolerance [109]. Altogether, these studies do not exclude a direct role of adipocytes in NE regulation and metabolism as human adipocytes, but not mouse adipocytes, possess all the machinery for NE transport and degradation via MAO-A, and to a lesser extent MAO-B [105,110,111]. In addition, the respective roles played by MAO-A or MAO-B in adipose tissue are still unclear as another study demonstrated that selective inhibition of MAO-B with selegiline conferred protection in the rat model of diet-induced obesity by decreasing adiposity as well as inflammation [112]. In conclusion, MAOs inhibition could represent a potential therapeutic strategy in adipose tissue through enhanced NE-induced lipolysis and putative reduction of the pro-inflammatory state of macrophages.

• **Role of monoamine oxidases in liver steatosis**

The role of MAOs has been also studied in obesity-related complications. Metabolic dysfunction-associated steatohepatitis (MASH) corresponds to the accumulation of fat in the liver. MASH is a complication of obesity, dyslipidemia and diabetes with a growing incidence in western countries. Interestingly, in a model of HFD in mice, administration of the non-selective MAO-A/MAO-B inhibitor phenelzine normalized metabolic alterations, including subcutaneous and hepatic fat, hypertriglyceridemia, insulin resistance and markers of oxidative stress [113]. Remarkably, a reduction of hepatic steatosis together with normalization of transaminase levels was observed, which could contribute to the amelioration of liver function. Indeed, a reduction of the HFD-induced leukocyte infiltration and the pro-inflammatory state (IL-6 and TNF-α) was also evidenced in this model [113]. The beneficial effects of MAO inhibition in liver steatosis were further demonstrated with selective inhibitors. Mice treated with the MAO-B inhibitor selegyline showed decreased body weight and fat mass, decreased circulating levels of cholesterol and triglycerides and decreased hepatic fat accumulation when given high-fat diet for 6 weeks [114]. Interestingly, reduction in oxidative stress and pro-inflammatory cytokines IL-6, IL-1β and TNF-α were found in HFD-mice treated with selegyline [114] and also deprenyl, another MAO-B irreversible inhibitor that decreased hepatic triglycerides and cholesterol [115]. *In vitro*, application of deprenyl on HepG2 cells inhibited triglyceride and cholesterol synthesis, while increasing cholesterol clearance [115]. This point toward hepatocytes as a main mediator of MAO-mediated lipotoxicity. Indeed, based on another work by Fu et al., HepG2 cells and primary hepatocytes incubated with palmitic acid (PA) engage a full serotoninergic response, leading to *de novo* lipogenesis, formation of lipid droplets and pro-inflammatory cytokine generation [116]. This provides one explanation on how MAO activity could be augmented through increased substrate availability in the liver. Mechanistically, they observed an intracellular 5-HT synthesis in response to PA or high glucose that leads to local activation of membrane serotoninergic 5-HT_2_ receptor mediating lipogenesis [116]. In parallel, intracellular 5-HT is degraded intracellularly by MAO-A, leading to ROS production, NF-κB activation and inflammatory response [116]. Finally, they observed that T2DM mice had increased levels of 5-HT in liver with upregulation of serotoninergic synthesis enzymes and 5-HT2 receptors. Blockade of 5-HT synthesis or 5-HT_2_ receptors prevented hepatic steatosis, inflammation, hyperglycemia and dyslipidemia *in vivo* [116]. Although this study provides an explanation on how hepatocyte MAO-A could be activated in the liver through local 5-HT synthesis and secretion, it remains uncertain how MAO-B is activated and regulates liver steatosis since 5-HT is not a preferential substrate for this isoform. Also, the importance of other cell types (macrophages, fibroblasts) in MAO-mediated effects were not studied in this context of liver steatosis.

• **Role of monoamine oxidases in atherosclerosis**

Another frequent complication of metabolic diseases is atherosclerosis, which corresponds to the gradual build-up of plaques in the artery walls, composed of fats, cholesterol, calcium and other substances. A pro-inflammatory environment that contribute to all stages of the disease characterizes atherosclerosis: initiation, progression and ultimately, thrombotic complications. Both MAO-A and MAO-B have been shown to be upregulated in atherosclerotic plaques of humans and ApoE-deficient mice [115]. However, only deprenyl treatment (MAO-B) but not clorgyline (MAO-A) reduced atherosclerosis in ApoE-deficient mice fed a cholesterol diet [115]. Most interestingly, MAO-B inhibition with deprenyl also reduced ROS, adhesion molecules, inflammatory cytokines and macrophages infiltration in atherosclerotic plaques. Endothelial dysfunction represents the earliest modification in the build-up of plaques and Tian et al. studied the specific effects of MAOs on endothelial cells [117]. They found that MAO-B was upregulated in HUVECs or human aortic endothelial cells exposed to palmitic acid (500 μM) for 24 h, while no effect was observed in smooth muscle cells [117]. MAO-B silencing or selegiline treatment reduced ROS burden in HUVECs incubated with palmitic acid, and inhibited transcriptome signaling pathways related to apoptosis and inflammation. Finally, treatment of ApoE−/− mice with selegiline in the model of HFD significantly decreased plaque area and inflammation [117], confirming the findings of Wang et al. This is in line with the observation that MAO-B is the predominant isoform in mammary arteries harvested from diabetic patients with coronary heart disease [118]. However, inhibition of either MAO-A or MAO-B in this model significantly improved endothelial relaxation [118]. In another study performed on mouse aortic rings, angiotensin II or LPS increased both MAO-A and MAO-B expressions which resulted in enhanced production of H_2_O_2_ and impaired relaxation The application of a MAO-A selective or a MAO-B selective inhibitor partially restored endothelium-dependent relaxation while combined inhibition showed additive effect [119]. *In vivo*, MAOs were of general importance in the control of endothelium-dependent relaxation in diseases conditions induced by Angiotensin II and LPS in mice [119]. Further studies evaluated the role of endothelial cells or macrophages in response to oxidized low density lipoprotein (oxLDL), which is a major mediator of leucocytes recruitment in atherosclerosis or palmitic acid (PA). HUVECs incubated with oxLDL or PA had increased levels of 5-HT, Tph1 and MAO-A leading to increased ROS production, NF-κB activation and inflammatory cytokines in the culture media [66]. Additionally, THP-1 cell-derived macrophages exposed to oxLDL or PA upregulate a full 5-HT system with the 5-HT synthases Tph1 and AADC, the 5-HT_2A_ receptor and MAO-A. They provide evidence that MAO-A-mediated ROS production in macrophages leads to NF-κB activation and the release of inflammatory cytokines [66]. Intriguingly, another study also point toward a role for MAO-A in atherosclerosis, but through NE degradation in atherosclerosis [120]. They used renal denervation as a strategy to reduce sympathetic tone in the model of ApoE^−/−^ HFD feeding in mice. They observed that renal denervation ablated peripheral sympathetic nerves and decreased NE levels in the circulation. RDN retarded the development of atherosclerotic plaques with potent reduction of mitochondrial ROS, inflammation and macrophages/foam cells, but no effect on hyperlipidemia [120]. On human aortic endothelial cells (HAECs), the authors demonstrated that MAO-A was the main mediator of NE-stimulated ROS production, mitochondrial dysfunction and NF-κB activation, leading to the release of atherogenic and pro-inflammatory molecules [120].

At present, future studies will be needed to decipher how serotoninergic system and sympathetic tone act in synergy during the development of metabolic diseases by using more specific approaches. Also, as previously observed in other tissues, the respective functions of MAO-A versus MAO-B remain unclear. Nevertheless, these novel and unrecognized roles for MAOs in dyslipidemia and hepatic steatosis could open new avenues for anti-inflammatory and anti-oxidative treatments.

### 5.2 Role of MAOs in the suppression of antitumor immunity

ROS are important inducers of tumorigenesis through the stimulation of proliferation, the induction of metabolic reprogramming that provides necessary nutrients to sustain high growth and the promotion of endothelial-to-mesenchymal transition, migration and angiogenesis [121]. Thus, as important sources of cellular ROS, MAOs were recently identified as major regulators of tumorigenesis and metastasis in different types of cancer [122]. The role of MAO-A in tumorigenesis was first discovered in the context of prostate cancer [123]. Wu et al. demonstrated that aggressive prostate cancers had increased MAO-A expression, and not MAO-B, and that knockdown of MAO-A reduced tumor growth and metastasis *in vivo* through HIF-1α and epithelial-to-mesenchymal transition inhibition [123,124]. Since then, the role of MAOs, and particularly MAO-A, has been extended to lung cancer [125], glioblastoma [126], colorectal cancer [127] or gastric cancer [128] and MAO inhibitors are currently in development to be used in combination with classical chemotherapeutic drugs [129].

As cancer tumorigenesis and metastasis are strongly associated with inflammation, recent studies have established a close link between MAO-A and pro-inflammatory mediators. Wu et al. observed that MAO-A levels were elevated in human bone metastatic prostate tumors compared to normal prostate or primary prostate tumor. *In vivo*, inoculation of prostate cancer cells overexpressing MAO-A led to increased bone metastasis in mice, while cells with MAO-A genetic silencing delayed the onset of bone metastasis and extended mouse survival [124]. Interestingly, they identified a particular MAO-A-dependent paracrine signaling pathway in metastatic prostate cancer cells through the secretion of Sonic Hedgehog Signaling Molecule (shh). In turn, Shh stimulated bone microenvironment to produce RANKL, IL-6 and TGFβ, promoting tumor seeding and colonization [124]. On the other hand, inflammatory mediators released by cancer cells also have an influence on MAO expression. The cytokine IL-13 is known to increase aggressiveness of colorectal tumors by promoting EMT, migration and invasion [130]. In their study, Dhabal et al. demonstrated that IL-13 signaling pathway was involved in the regulation of MAO-A expression in A549 lung epithelial carcinoma cells, promoting ROS production, EMT, migration and metastasis [125]. Importantly, the tumorigenic effects of IL-13 were blocked by the MAO-A inhibitor moclobemide [125]. In cholangiocarcinoma, on the other hand, MAO-A expression is suppressed, and the reduced level of MAO-A increases cancer invasiveness [131]. As a result, it appears that MAO-A regulation and function varies amongst cancer types.

**Fig. 3:**
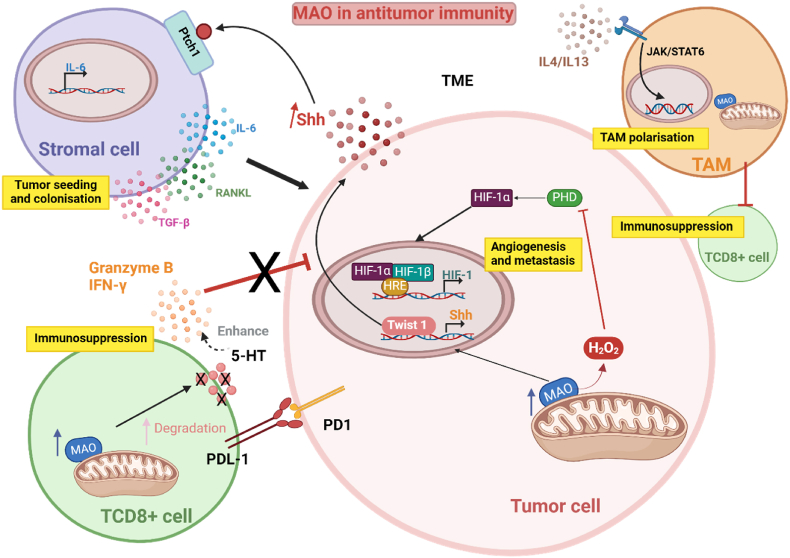
Role of MAOs in the suppression of antitumor immunity. Monoamine oxidases (MAOs) appear to regulate various mechanisms, depending on the type of cancer. On the one hand, increased levels of MAO-A can lead to increased production of ROS, which in turn inhibit Prolyl hydroxylase (PHD), a key regulator of hypoxia-inducible factor alpha (HIF-α), which, by binding to HIF-β, translocates to the nucleus and enables transcription of target genes involved in angiogenesis, tumor growth and metastasis. In addition, MAO-A is involved in paracrine Sonic Hedgehog (Shh) signalling, which, by binding to the Ptch1 receptor of surrounding stromal cells, enables secretion of RANKL (receptor activator of nuclear factor-κB ligand), IL-6 and TGFβ, promoting tumor seeding and colonization. Overexpression of MAO-A may also lead to polarization of macrophages into tumor-associated macrophages (TAMs), via IL-4 and IL-13, inducing the JAK-STAT6 signaling pathway. This TAMs polarization is thought to contribute to the immunosupression of TCD8+ lymphocytes, which are involved in anti-tumor defence. Finally, the overexpression of MAO-A in TCD8+ lymphocytes also enables immunosupression through the increased degradation of serotonin, an important player in TCD8+ activation and hence the secretion of cytotoxic molecules such as granzyme B and interferon y (IFN y) involved in the degradation of cancer cells.

In addition to cytokine regulation, some very recent studies discovered a new mechanism by which MAO-A regulated antitumor immunity [100,132]. Tumor-associated macrophages (TAMs) are important players in cancer immunity as they can regulate the cytotoxic function of T cells. The polarization of TAMs toward an immunosuppressive phenotype can inhibit the elimination of tumor cells by T cells and is an important obstacle for cancer immunotherapy. In their study, Wang et al. observed an upregulation of MAO-A expression and immunosuppressive markers in TAMs of mice inoculated with B16-OVA melanoma tumors. They demonstrated that MAO-A acted as an autonomous factor promoting the polarization of TAMS toward an immunosuppressive mechanism, via ROS production and activation of IL-4/IL-13-induced JAK-Stat6 pathway [100]. *In vivo*, they showed that MAO-A KO mice or WT mice inoculated with bone marrow-derived macrophages from MAO-A KO mice, had decreased tumor growth following injection of B16-OVA cells. Consistently, MAO-A knock-down led to reduced levels of immunosuppressive genes and increased levels of pro-inflammatory cytokines in TAMs. Finally, they demonstrated the synergistic effect of a combined treatment with PD-1/PD-L1 and phenelzine (a mixed MAO-A/MAO-B inhibitor) on the inhibition of tumor progression in B16-OVA (melanoma) and MC38 (colon cancer) models [100]. In another study, Wang et al. demonstrated that MAO-A gene was also upregulated in CD8^+^ T cells and directly regulated their cytotoxic function in the B16-OVA model [132]. Tumor-infiltrating CD8^+^ cells in MAO-A KO mice showed enhanced activity with higher levels of cytotoxic molecules (granzyme B, IFN-γ). Interestingly, the mechanism of action of MAO-A was not related to ROS production in CD8^+^ T cells. Instead, inhibition of MAO-A increased the levels of serotonin, which acted as an autocrine factor enhancing T cell activation through 5-HTRs surface receptors. Finally, clinical data correlation studies associated intratumoral *MAOA* expression with T cell dysfunction and decreased patient survival in a broad range of cancers [132]. Altogether, these novel discoveries identify MAO-A as an immune checkpoint through direct regulation of tumor macrophages and lymphocytes (Fig. 3).

### 5.3 Putative role of MAOs in the regulation of cardiac inflammation

Sterile inflammation occurs as a secondary response to myocardial damage from ischemia or other causes of HF. The levels of cytokines and chemokines are increased in patients with HF and represent independent risk factors of mortality [133]. Pro-inflammatory mediators in the heart originates from various sources such as cardiomyocytes, endothelial cells, cardiac fibroblasts and resident macrophages. Additionally, immune cells such as neutrophils and macrophages will respond to cytokines by producing more cytokines/chemokines under pathological conditions, which initiates a vicious cycle, resulting in chronic heart inflammation [133]. Although multiple reports have indicated that inflammation plays a significant role in cardiac diseases, the exact mechanisms that trigger the onset and sustain chronic inflammation remain unclear.

The relative expressions of cardiac MAO-A and MAO-B are species-dependent and MAO-A is highly predominant over MAO-B in the rat heart, while the opposite occurs in mice [58]. In humans, both enzymes are expressed at similar levels in the heart [134]. Accumulating evidence demonstrates the involvement of MAOs in cardiac disorders such as ischemia-reperfusion injury (I/R) [135,136], post-myocardial infarction remodelling [74], arrhythmias [137], pressure overload-driven HF [75,80,138], diabetic cardiomyopathy [139,140] and doxorubicin toxicity [141]. While physiological MAO activity may support the regulation of catecholamines and 5-HT concentrations in the heart to fine-tune the intracellular sarcoplasmic reticulum β1-adrenergic receptor signaling [142], overactivation of these enzymes in stress conditions is detrimental through the excessive production of ROS and/or aldehydes [58]. This overactivation of MAOs at the onset of cardiac diseases has been correlated to the upregulation of the enzymes at the mRNA or protein level and also to the overload of substrates such as 5-HT and NE [58]. To date, the deleterious effects of cardiac MAO rely on specific signaling mechanisms related to hypertrophy, senescence, cell death, autophagy dysregulation, epigenetic modifications and mitochondrial alterations [67,143]. Now, some recent evidences suggest that inflammation could be a potential mechanism of action of MAOs in the heart.

• **Consequences of MAO-A overexpression in cardiomyocytes**

In order to better understand the consequences of MAO-A overactivation observed in HF, mice with forced expression of MAO-A in cardiomyocytes (MAO-A Tg) were generated [83]. These mice displayed accumulation of oxidative stress and aldehydes in the heart at 6 weeks, followed by mitochondrial damage, cardiomyocyte senescence and necrosis, which led to premature HF and death at 6 months [74,83,144]. Some significant enrichment of Gene Ontology pathways related to “Inflammation, chemotaxis, Immune response and cytokine signaling” was observed by transcriptomic analysis in MAO-A Tg mice [83]*.* Interestingly, the cardiac microenvironment was profoundly modified in these mice before the onset of HF, as recently shown by Martini et al. [93]. MAO-A Tg hearts displayed an accumulation of CD45^+^ cells. MAO-A-mediated ROS production in cardiomyocytes induced premature senescence of stromal mesenchymal stem cells and promoted the increase of CCR2^+^ cardiac macrophages and of proinflammatory cytokine expression [93]. Hence, this highlights that some specific cross-talk between cardiomyocytes and stromal cells regulate inflammation process in the heart in response to MAO-A activation. Due to the global alteration of mitochondrial function in this model and the apparent oxidation observed on mtDNA and cardiolipin [74,83], it is possible that the modifications of the microenvironment involve the release of mito-DAMPs, although this possibility will need further investigations.

• **Role of MAOs and inflammation in ischemic heart diseases**

The overload of substrates is a prominent feature of I/R injury, fuelling MAOs activity with the liberation of 5-HT from activated platelets [145] or the liberation of NE by sympathetic nerve terminals [146,147]. More recently, mass spectrometric approaches dedicated to the cardiac “aminome” unexpectedly identified N^1^-methylhistamine (NMH), the methylated product of histamine, as a major substrate of MAO-B during ischemia-reperfusion in Langendorff-perfused hearts and in right ventricular failure mouse model [147,148]. Histamine could be released under stress conditions by sympathetic neurons or mast cells, which lie adjacent to blood vessels and between cardiomyocytes [149]. In addition, some studies suggested that 5-HT could be formed inside the heart, probably by cardiomyocytes themselves [150]. In this context, some early studies have suggested a link between MAOs and inflammatory markers. In rats, I/R injury can be prevented by the administration of the MAO inhibitors pargyline or clorgyline, leading to decreased ventricular damage and oxidative stress and also decreased infiltration of leucocytes (macrophages, neutrophils) as shown by myeloperoxidase (MPO) activity [135]. In mice, both global MAO-A KO and cardiomyocyte-specific MAO-B KO showed protection against ROS accumulation and ventricular damage following I/R, suggesting that both isoforms contribute to monoamine catabolism and H_2_O_2_ formation in the heart [74,151,152]. Another study tested whether MAO inhibition could enhance myocardial recovery in rats after heart transplantation and myocardial infarction, as perioperative myocardial infarction occurs in 1–2% of patients after cardiac surgery [153]. Interestingly, moclobemide treatment decreased some inflammatory markers in this model. Further validation will be needed in future studies on the importance of MAO in inflammation during I/R damage.

• **Role of MAOs and inflammation in diabetic cardiomyopathy**

Diabetic cardiomyopathy is a condition characterized by high glucose and cytotoxicity leading to diastolic dysfunction, myocardial stiffness and fibrosis. The underlying mechanism is complex and involves mitochondrial dysfunction, increased oxidative stress and inflammation. Indeed, sustained NLRP3 activation and cytokines IL-1β and IL-18 can exacerbate tissue dysfunction and fibrosis in diabetic cardiomyopathy [154]. MAO-A was recently identified as an important molecular player in streptozotocin-induced cardiomyopathy as its inhibition with clorgyline reduced ventricular dysfunction in rats [140]. In an interesting study, Deshwal et al. further elucidated the mechanisms of action of MAO-A in diabetic cardiomyopathy [139]. They demonstrated that in cultured cardiomyocytes, a combination of high glucose and IL-1β (HG + IL-1β) upregulated MAO-A-dependent ROS production, which led to opening of the permeability transition pore (PTP), the reduction of mitochondrial membrane potential and ER stress. On the other hand, MAO-A was not involved in the activation of NLRP3 induced by HG + IL-1β in cardiomyocytes [139]. *In vivo*, they further demonstrated that MAO inhibition with pargyline ameliorated lipid peroxidation, ER stress, fibrosis and diastolic dysfunction induced by streptozotocin. A reduction in mast cells degranulation was also observed in hearts treated with pargyline, which is a potential contributor of fibrosis and cytokines release [139]. According to this study, mast cells contain dopamine, 5-HT and histamine, which might fuel MAO activation in the context of diabetes.

In conclusion, although the respective contributions of MAO-A versus MAO-B in the heart are still unclear and may present some redundancy due to substrate overlap, these enzymes could participate in the exacerbation of inflammatory response in different pathological contexts.

### 5.4 Role of MAO-B in rheumatoid arthritis

Although MAOs are relevant sources of ROS and can be upregulated by pro-inflammatory signals, their roles in autoimmune disorders is just beginning to be unraveled. Rheumatoid arthritis (RA) is a chronic autoimmune inflammatory disease characterized by joint swelling and pain, along with cartilage damage and bone resorption [155,156]. Synovial membrane inflammation is due to the infiltration of leukocytes, innate and adaptive immune cells that release cytokines and chemokines. The specific organs affected in RA include the skin, eye, lung, kidney, and brain. Intriguingly, an early clinical report indicated that some patients treated for their depression with MAO inhibitors (tranylcypromine, phenelzine and isocarboxazid) reported improvement of joint pain due to rheumatoid arthritis [157]. In a recent study, Won et al. reported that fibroblasts-like synoviocytes of RA patients that were treated with TNF-α showed significant elevation of MAO-B mRNA and protein, but not MAO-A [158]. A concomitant elevation of oxidative stress and the inflammatory mediator IL-6 was observed together with MAO-B. Next, the authors tested the inhibition of MAO-B with a new selective and reversible inhibitor KDS2010 on the model of collagen-induced arthritis (CIA). While MAO-B was upregulated in the joints of CIA mice, MAO-B inhibitor decreased the arthritis clinical score, inflammatory infiltration and cytokine TNF-α levels [158]. This study demonstrated for the first time that MAO-B was involved in joint inflammation in RA and also in the cognitive impairment frequently observed in patients. In a similar study performed in rats, the administration of selegiline ameliorated RA development induced by a combined injection of collagen + LPS, with decreased levels of oxidative stress, TNF-α and IL-6 and reduced synovial cell proliferation [159]. Another model was tested in mice with the injection of calcium pyrophosphate dehydrate crystals (CPP) into the ankle joints to induce inflammatory arthritis [160]. Interestingly, MAO-B inhibitors rasagiline or safinamide reduced ankle swelling at 48 h and dampened the expression levels of cytokines and chemokines. In addition, in macrophages challenged with CPP, the increase of ROS levels, the mitochondrial depolarization and NF-κB activation were all prevented by rasagiline and safinamide [160]. Thus, MAO-B activity is required for the activation of macrophages and the initiation of inflammatory response induced by CPP.

### 5.5 Role of MAO-B in obstructive pulmonary disease (COPD)

Chronic obstructive pulmonary disease (COPD) is a chronic inflammatory disease that causes obstruction of airflow from the lungs due to long-term exposure to irritating gases or particles, most often from cigarette smoke (CS). In lung mucosal tissue, CS increases oxidative stress and inflammation through the involvement of different types of immune cells such as macrophages, neutrophils and T cells. Other lung cells such as epithelial cells can also produce inflammatory mediators [161]. Interestingly, in human airway epithelial cells, cigarette smoke (CS) application led to upregulation of MAO-B activity [162]. Inhibition of MAO-B with selegiline reduced oxidative stress induced by CS and attenuated the release of IL8 through NF-κB inhibition [162]. Later on, treatment of rats exposed to CS for 7 days with selegiline normalized lung oxidative stress and inflammatory mediator levels, without changing the absolute number of inflammatory cells [163]. Again, NF-κB but also MAPK activations were reduced by selegiline, as previously observed *in vitro*. These observations will need to be confirmed in a new model of long-term exposure to CS in order to see the effect of MAO-B inhibition on lung emphysema [163].

## 6 Conclusion and studies in perspective

**Fig. 4:**
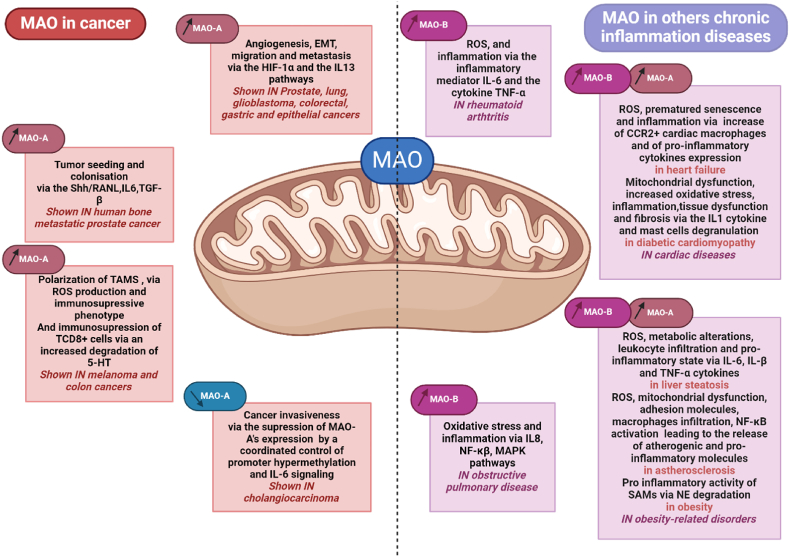
Pathophysiological evidences on the role of MAOs in chronic inflammation: cancers and other chronic diseases. Comparison of the regulation and potential functions of MAOs via different mechanisms in different types of cancers and other chronic inflammatory diseases.

Low-grade inflammation underlies the development of various chronic diseases and recent advances have put forward the role of mitochondria and mito-DAMPs in sustaining pro-inflammatory cytokines and chemokines. MAOs are at the nexus of neurohormonal signaling, mitochondrial dysfunction and ROS/aldehyde generation, representing key targets for modulating inflammation. Indeed, MAOs have been shown to regulate the onset and progression of many chronic diseases, but until now, their impact on inflammatory processes has often been overlooked. Based on recent studies using genetically-modified mice or selective MAO inhibitors, there is now strong evidences that MAOs display either pro-inflammatory (obesity, cardiovascular diseases, pulmonary diseases and rheumatoid arthritis) or anti-inflammatory (Cancer) effects in preclinical models of chronic diseases (Fig. 4). However, there are still some limitations associated with these findings. One of them is the lack of comparative analysis between males and females in preclinical models described above. Some important gender differences exist for MAOs in humans, as exemplified by the higher plasma and brain MAO activities in women compared to men [164,165]. Among the possible explanations is the observed modulation of MAO-A and MAO-B promoter activities by estrogens [166] or testosterone [167]. This sex-dependent effect of MAOs is well illustrated in the model of cardiac ischemia-reperfusion injury. As shown by Heger et al., female mice are more resistant to I/R damage than males [168]. In addition, while MAO-B genetic deletion is protective in males, there is no such effect in females [168]. In humans, the Brunner syndrome, corresponding to a deletion in MAO-A gene, is associated with borderline mental retardation and aggressive behavior in males but not in females [169]. Whether this is due to incomplete X-inactivation in females or to other mechanisms is still to be determined. Hence, gender could have major consequences on the susceptibility to diseases and the response to treatment, that will need to be taken into account in future studies.

Another caveat in the field is the lack of comprehension on the respective roles of MAO-A versus MAO-B in some chronic inflammatory diseases. In the heart, blockade of one or another isoform shows redundant effect, indicating that they might have some overlapping functions. Also, the distribution of the two isoforms is tissue-specific and species-specific. Therefore, the translation to humans will need to take into account some particular differences compared to rodents. Despite this complexity, accumulating evidence now supports the relevance of targeting these enzymes to modulate inflammation and immune cells function in chronic inflammatory diseases and cancer [170]. MAO inhibitors (MAOi) have already proven to be effective for the management of a variety of mental disorders such as depression, Parkinson's disease and Alzheimer's disease [51]. In the case of Parkinson's disease, the mechanisms of action, pharmacodynamics and pharmacokinetics of MAOi (Selegyline, Rasagiline) are well established through the blockade of mitochondrial ROS generation and mitochondrial dysfunction. In this particular case, MAOi have been suggested to play a role in the associated neuro-inflammation [171]. In cancer, some clinical trials are currently ongoing to assess the beneficial role of phenelzine (MAO-A/MAO-B inhibitor) in prostate tumorigenesis and metastasis. In other pathologies, there is a need to translate pre-clinical findings into human-based clinical studies in the future. In conclusion, designing drugs to modulate the activity of MAOs might lead to therapeutic avenues to treat many chronic inflammatory diseases.

## Funding

This work was supported by grants from the Institut National de la Santé et de la Recherche Médicale (INSERM), 10.13039/501100001665Agence Nationale de la Recherche (ANR-19-CE14-0038-01), Fédération Française de Cardiologie (FFC), Région Pays de la Loire. CGL and ORB are supported by grants from AFM-Téléthon (#25002 and #25034, DYNAMYO).

## CRediT authorship contribution statement

**Lise Beucher:** Writing – review & editing, Writing – original draft. **Claudie Gabillard-Lefort:** Writing – review & editing, Writing – original draft. **Olivier R. Baris:** Writing – review & editing, Writing – original draft. **Jeanne Mialet-Perez:** Writing – review & editing, Writing – original draft, Validation, Conceptualization.

## Declaration of competing interest

None.

## Data Availability

No data was used for the research described in the article.
